# Automated measurement of detectability index in CT imaging: Development and validation

**DOI:** 10.1002/acm2.70352

**Published:** 2025-11-14

**Authors:** Choirul Anam, Ariij Naufal, Zaenal Arifin, Eko Hidayanto, Evi Setiawati, Fajar Arianto, Toshioh Fujibuchi, Geoff Dougherty

**Affiliations:** ^1^ Department of Physics Faculty of Sciences and Mathematics Diponegoro University Semarang Central Java Indonesia; ^2^ Department of Health Sciences Faculty of Medical Sciences Kyushu University Fukuoka Japan; ^3^ Applied Physics and Medical Imaging California State University Channel Islands Camarillo California USA

**Keywords:** detectability index, model observer, task‐based image quality

## Abstract

**Purpose:**

The purpose of this study is to develop software for measuring the detectability index (d′) automatically from ACR 464 CT phantom images.

**Method:**

Software for measuring d′ automatically was developed with Python 3.9.13 using the PyQt5 graphical user interface (GUI) as part of the IndoQCT platform. The task‐transfer function (TTF) and noise power spectrum (NPS) were automatically measured to obtain spatial resolution and noise texture information. The task function was defined with a Gaussian and flat types, a matrix size of 300 pixels, a pixel size of 0.05 mm, and a contrast of 15 HU. The task object diameter was set to 5 mm for the tube current and kernel type variations, and ranged from 1 to 15 mm for the object diameter variation. The task object contrast ranged from 1‐29 HU for the object contrast variation. Each dataset was evaluated in terms of the d′ using the non‐pre‐whitening (NPW) model observer. Images of an ACR 464 CT phantom scanned using a GE Revolution EVO scanner with tube currents of 80, 100, 120, 140, 160, and 200 mA and kernel types of Standard, Edge, Lung, and Soft were used for evaluation. The results of our developed software were compared with ImQuest results.

**Results:**

In general, our developed software produced d′ values that were in strong agreement with ImQuest across all tested variations and both task function types (Gaussian and flat). For tube currents, an increase in tube current consistently increased the d′ value (*r* = 0.98). Analysis of kernel types showed that the Standard kernel yielded the highest detectability, while the Lung kernel yielded the lowest. For variations in task object diameter and contrast, larger diameters and higher contrasts increased detectability following exponential (*R* ^2^ > 0.99) and linear trends (*R* ^2^ = 1), respectively. Across all variations (kernel, object diameter, and contrast), the correlation between IndoQCT and ImQuest was exceptionally strong (*r* > 0.98), validating the performance of the developed software.

**Conclusion:**

Software to automatically measure the d′ has been successfully developed. It is easily accessible with a straightforward, fast, accurate, and intuitive workflow.

## 1 INTRODUCTION

Advances in computed tomography (CT) imaging technology has made it one of the most widely used modalities for diagnostic purposes.^1^ Compared to other medical imaging modalities, CT provides advantages in detecting subtle lesions with high details.^2^, ^3^ However, achieving good detectability requires optimal configuration of both basic image quality parameters and operational settings. So far, medical images have been characterized physically using various parameters such as the spatial resolution (measured with a line‐pair phantom or modulation transfer function (MTF)),^4^, ^5^ noise level (calculated from the standard deviation in a homogeneous region), the noise power spectrum (NPS)^6^), and signal‐to‐noise ratio (SNR).^7^ These parameters are routinely measured in quality control (QC) programs, acceptance tests, and other evaluations. These parameters are certainly useful for determining the reliability of a CT imaging system. However, they do not directly correlate with the detectability index of low‐contrast lesions.^8^ Therefore, for operational optimization, these parameters cannot be used directly.

To achieve operational optimization in CT imaging, it is necessary to evaluate lesion detectability using a large number of images with various predefined input parameters to obtain the optimal level of disease detection accuracy. In this process, radiologists are asked to assess many images obtained from scans with specific input parameters. The radiologists' assessments are performed using the receiver operating characteristic (ROC) curve^9^ or percentage correct (PC) in n‐alternative forced‐choice (n‐AFC) experiments.^10^ Of course, it is not possible to repeatedly scan patients to vary the input parameters. A dedicated phantom with low‐contrast objects is needed to evaluate the detectability of low‐contrast lesions.^11^

Three main components are required to obtain the detectability index (d′). First, the availability of a dedicated phantom that represents a specific disease (task‐based phantom). The presence of such phantoms remains a significant challenge in several healthcare facilities worldwide, and particularly in Indonesia. Second, a large number of phantom image datasets must be acquired to produce statistically reliable analysis. It is important to note that for operational protocol optimization, analysis must be conducted across various scan configurations to identify the combination that provides the maximum benefit for a specific case. From an efficiency perspective, performing a large number of scans for this purpose is both costly and exhausting. Third, evaluating a large dataset requires commitment from human observers.^12^

To address these challenges, many mathematical models have been introduced as surrogates for human observers, known as model observers (MO).^13^ Several commonly used MOs include the non‐pre‐whitening (NPW) matched filter,^14^ NPW with an eye filter (NPWE),^15^ and the channelized Hotelling observer (CHO) with various filter types, such as Gabor, Difference of Gaussian (DoG), Difference of Mesa (DoM), and Laguerre–Gauss (LG).^16^, ^17^ These models have been proven to produce reading patterns and accuracy levels similar to those of human observers (HO) with varying degrees of accuracy and precision.

Although MOs can effectively surrogate human observers, they still do not resolve the issues of requiring task‐based phantoms and large datasets. Many efforts have been made to propose techniques for obtaining the d′ using only a standard phantom for quality control (QC) and a limited number of scans. In this approach, the d′ is computed through a complex model utilizing well‐established physical parameters such as the noise power spectrum (NPS) and the modulation transfer function (MTF) or task‐transfer function (TTF). With appropriate task object models and model observers, the d′ can be determined.^14^, ^18^

Specialized software is required to obtain the d′ value easily and quickly. Several software have been developed for this purpose, such as ImQuest^14^ and iQMetrix‐CT.^19^ These tools typically require manual user intervention to define regions of interest (ROI) for calculating TTF and NPS. To streamline this workflow and enable rapid, repeatable analysis of large datasets, we developed software to fully automate the d′ calculation process. To ensure our automated method provides results consistent with established techniques, we validated the d′ calculations against ImQuest using data with variations in tube current, kernel type, task object diameter, and task object contrast level using ACR 464 CT phantom images.

## 2 MATERIALS AND METHODS

### 2.1 Internal structure of the software

The software was developed using Python 3.9.13 and the PyQt5 graphical user interface (GUI) framework. The software workflow was designed to be as simple and intuitive as possible to facilitate user interaction with its features. The key components for d′ calculations include the type of phantom and key image quality parameters (TTF and NPS). The software was integrated to IndoQCT,^20^ as a platform for analyzing CT image quality. The IndoQCT can be found at https://indosect.com/indoqct.

### 2.2 Phantoms and automated algorithms

**FIGURE 1:**
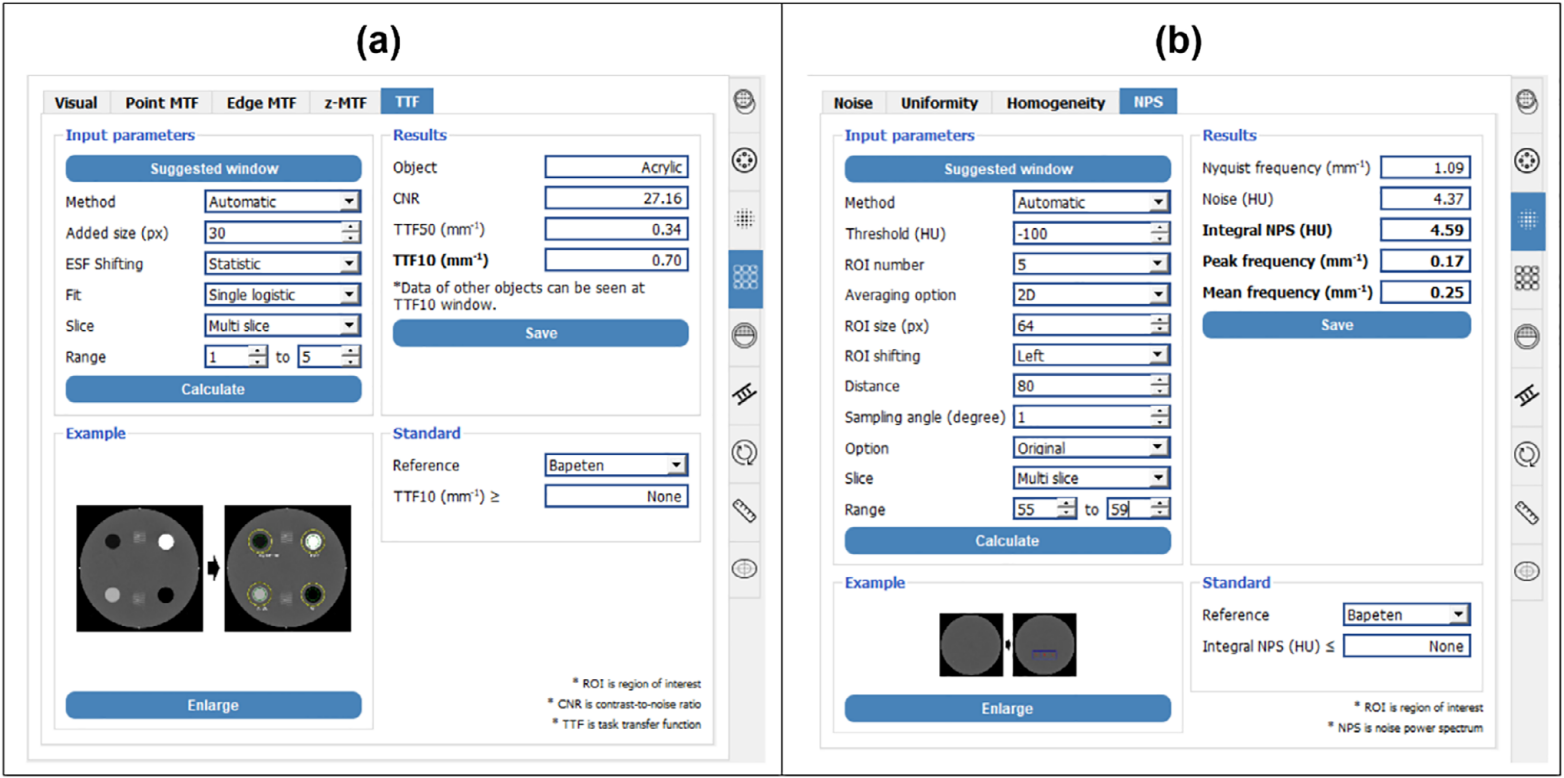
Screenshots of tab design for automatic measurement of image quality parameters using the ACR 464 CT phantom. (a) TTF and (b) NPS.

We have provided automated algorithms for measuring TTF and NPS for most commercially available CT phantoms, e.g. AAPM CT Performance phantom (model 610),^21^ ACR 464 CT phantom,^22^, ^23^ Catphan phantom (series 500‐604), and most of the built‐in CT image quality phantoms (Philips, GE, Siemens, Canon, and Neusoft). To measure a specific image quality parameter on a particular phantom type, users can select the available phantom, align the main viewer with the target image following the provided reference guide, and then proceed to the corresponding parameter tab for calculations. Figure 1 shows a screenshot of the GUI design for TTF and NPS measurements.

### 2.3 TTF measurement

**FIGURE 2:**
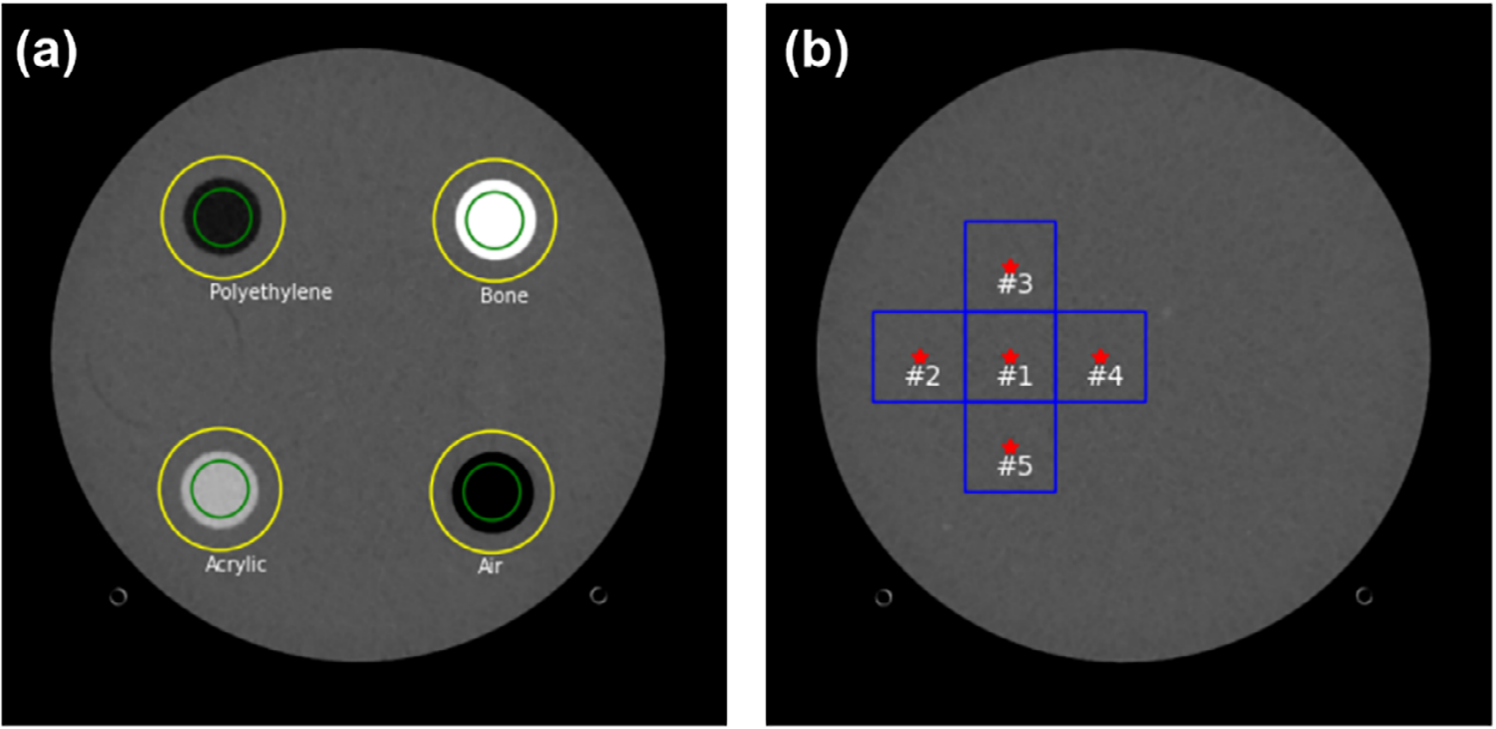
ROI positions for automatic measurement of key image quality parameters on image from ACR 464 CT phantom. (a) TTF of the four circular inserts, (b) NPS in the homogeneous area. For the TTF measurements, each insert has two ROIs, e.g. inner ROI (green) and outer ROI (yellow). Inner ROI indicates the border which is used to calculate the average CT number of the insert. The outer ROI indicates the border for calculating the average CT number and standard deviation of the background. Both average CT number (insert and background) were used to calculate CNR as a property of TTF.

Spatial resolution information can be obtained practically using TTF through an automated method (Figure 1a). The automated process begins by isolating the entire phantom from the surrounding air using a global threshold of −200 HU to create a binary mask. Within this masked region, the algorithm proceeds to segment the individual cylindrical inserts in Module #1 of the ACR 464 CT phantom (bone, polyethylene, air, and acrylic) by applying pre‐defined, material‐specific HU thresholds. For each segmented insert, the geometric centroid is calculated to precisely determine its central coordinates.^23^ Circular ROIs whose radius is equal to the insert radius plus 30 pixels are then placed on each insert to extract the edge spread function (ESF) response with an angular sampling of 10° inclination (Figure 2a). The ESF samples are phase‐aligned statistically and fitted using a single logistic fit to eliminate noise effects.^24^ The single logistic fit ensures that the edge profile remains strictly monotonic. The line spread function (LSF) is then obtained by differentiating the estimated ESF, and is subsequently transformed using the Fourier transformation to obtain the TTF (Equation (1)).

(1) $$TTF\left(f\right)=FFT\left(LSF\left(n\right)\right)=\left|\sum_{n=0}^{N-1}LSF\left(n\right)e^{-2\pi ifn/N}\right|$$

The frequencies at 50% and 10% of the TTF response are calculated, along with the contrast‐to‐noise ratio (CNR), which complements the contrast‐dependent spatial resolution characteristics. In this study, TTF measurements were performed on five consecutive slices.

### 2.4 NPS measurement

NPS was measured by placing multiple square ROIs with size of 64 × 64 pixels in a homogeneous region of the ACR 464 CT phantom located in Module #3.^25^ Since this module contains two point objects, the automated method shifted the ROI center to the remaining homogeneous area (left side) by default. Various ROI numbers were available in the automated method; in this study, we used five ROIs. NPS measurements were also conducted across five slices (Figure 2b). Once the ROIs were placed, the 2D NPS was calculated using Equation (2).

(2) $$\begin{array}{cc} & \mathrm{NP}S_{2D}\left(u,v\right)=\\ & \frac{\Delta x}{L_{x}}\frac{\Delta y}{L_{y}}\frac{1}{N_{\mathrm{ROI}}}\sum_{i=1}^{N_{\mathrm{ROI}}}{\left|\mathrm{FF}T_{2D}\left\{\mathrm{RO}I_{i}\left(x,y\right)-\bar{\mathrm{RO}I_{i}\left(x,y\right)}\right\}\right|}^{2} \end{array}$$

where *u* and *v* are the spatial frequencies in X and Y directions, Δx and Δy are the pixel sizes in the X and Y directions, FFT represents the Fourier transformation, $L_{x}$ and $L_{y}$ are the ROI dimensions in the X and Y directions, and $N_{ROI}$ is the number of ROIs.

The above process produced a 2D NPS in the frequency domain, averaged across all ROIs over the selected slice range. To simplify the representation of the 2D NPS, its 1D NPS was obtained by radially averaging the 2D NPS, providing a summary of the integrated NPS in HU, with maximum and mean frequencies in mm^−1^. These three parameters effectively summarize the noise texture characteristics. Additionally, an optional curve smoothing feature was available, using polynomial fitting of various orders.^26^

### 2.5 Detectability index

**FIGURE 3:**
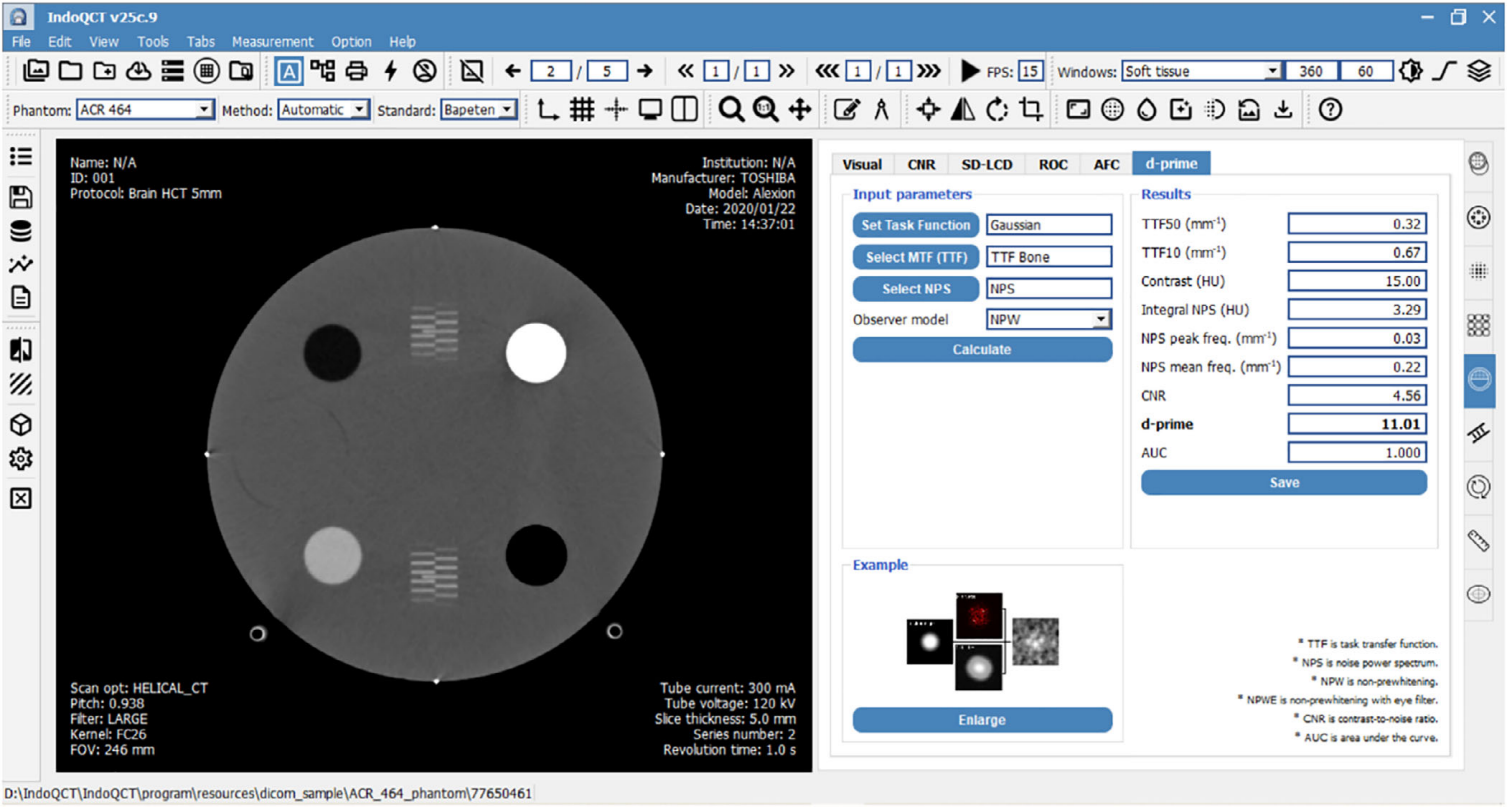
Graphical user interface for calculating detectability index (d′).

Figure 3 shows the GUI for calculating the d′. Several components must be gathered for this calculation, including TTF, NPS, task function definition, and model observer. Once the TTF was measured, the user can select the TTF of a specific material as the spatial resolution component (e.g., TTF from bone material). Additionally, if the phantom used did not provide multiple circular inserts, the resolution component can also be selected from point‐based MTF^27^ and edge‐based MTF.^28^ The NPS component can be selected in a similar manner to the spatial resolution component.

Task function definition was performed by configuring its parameters. The task function was defined as a circular nodule simulating two different clinical tasks: **Flat**, which represents a homogeneous circular nodule; and **Gaussian**, which simulates a hepatic nodule.^14^

In this study, the task function was set with a matrix size of 300 pixels, a pixel size of 0.05 mm, an object size of 5 mm, and a contrast of 15 HU. The task function ($W_{task}$) in the frequency domain was then mathematically defined according to Equation (3).

(3) $${\left|W_{task}\left(u,v\right)\right|}^{2}={\left|\Delta p^{2}.FFT_{2D}\left\{c\left(r\right)\right\}\right|}^{2}$$

where *u* and *v* are the spatial frequencies in the X and Y directions, Δp is the pixel size, $FFT_{2D}$ is the 2D Fourier transform of the task function, and *c* is the value of the task function at a radial distance *r*.

**FIGURE 4:**
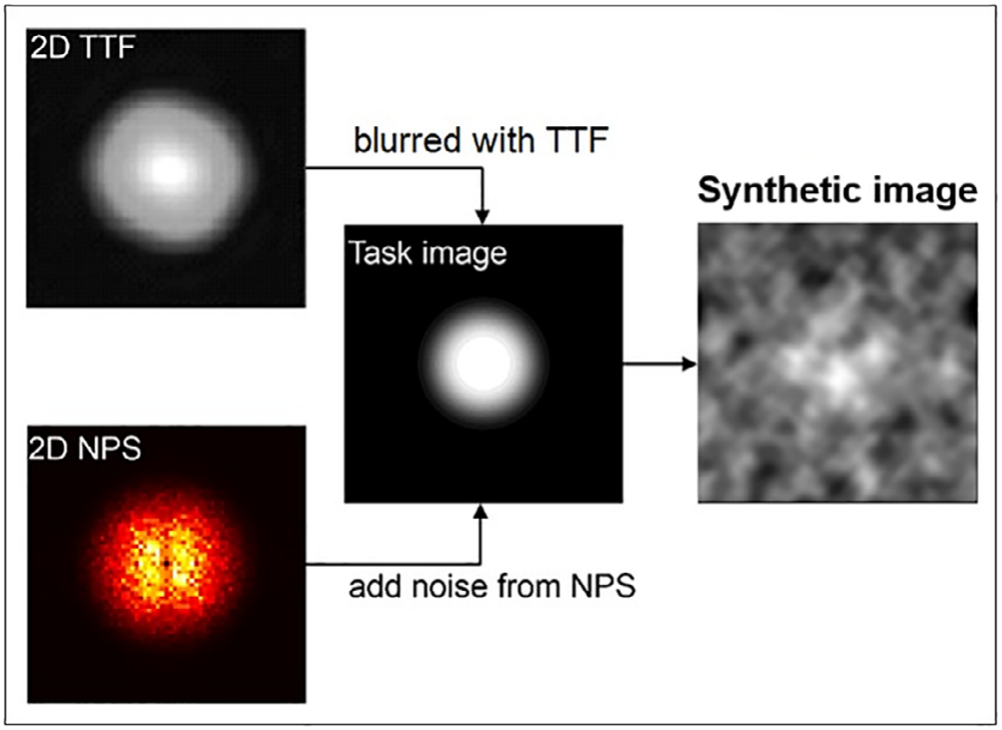
Illustration of the synthetic image generation from the task image that is blurred using 2D TTF and added with noise from 2D NPS. This synthetic image is a representation of a real image scanned with certain input parameters.

The generation of synthetic image is illustrated in Figure 4. This synthetic image is a representation of a real image containing task object scanned with certain input parameters. Since the frequency limits of TTF and NPS are much lower for $W_{task}$ with a pixel size of 0.05 mm, a resampling is necessary. Resampling process aims to match the measured TTF and NPS frequency grids with the task function frequency grid. Resampling is performed in 2D, with linear interpolation referring to the task function frequency grid, and a fill value of 0 for interpolation outside the data range. The images were blurred using 2D resampled TTF in the frequency domain. Finally, noise texture was added to the image in the spatial domain. The synthetic image is a visual representation of a nodule resembling a low‐contrast lesion with sharpness and noise texture similar to an actual scan image.

Once the synthetic image was generated, the next step was to calculate its detectability using a model observer. The software employed a model observer known as non‐pre‐whitening (NPW) to compute the d′.^14^ This model was based on the principle of template matching, where cases exist in either a signal‐present or signal‐absent condition. Additionally, several modifications of NPW were available, including NPW with an eye filter (NPWE) to more realistically simulate human visual characteristics, and versions incorporating internal noise (NPWi) to simulate the internal noise in human visual perception. Mathematically, d′_NPW_ was defined by Equation (4).

(4)

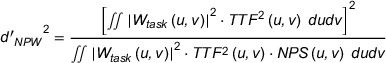




### 2.6 Data collection

We collected four types of variations for d′ measurement: tube current, kernel type, task function object diameter, and task function object contrast variations. For tube current and kernel type variations, we scanned the ACR 464 CT phantom using a GE Revolution EVO scanner under the conditions described in Table 1. For task function object diameter and contrast variations, we extracted the TTF of the bone insert and NPS data from the dataset at 140 mA tube current. The task object diameter was varied from 1 to 15 mm for variation of object diameter, and 1 to 29 HU for variation of object contrast: both were modelled for Gaussian and flat nodules.

**TABLE 1:** ACR 464 CT phantom scan parameters for tube current and kernel type variations.

Parameter	Tube current variation	Kernel type variation
Tube current (mA)	80, 100, 120, 140*), 160, and 200	160
Tube voltage (kV)	120	120
Slice thickness (mm)	1.25	1.25
Exposure time (ms)	0.8	0.8
FOV (mm)	235	235
Scan mode	Helical	Helical
Pitch	0.53125	0.53125
Reconstruction	FBP	FBP
Filter	Head	Head
Kernel	Standard	Standard, Edge, Lung, and Soft

*) Dataset from 140 mA tube current was used for variations of task function object diameter and contrast.

### 2.7 Statistical analysis and software comparison

To quantitatively validate the performance of the developed software, d′ results from the NPW model observer outputs were directly compared against those generated by ImQuest. The primary metric for this comparison was the Pearson correlation coefficient (*r*), which was used to assess the strength and direction of the relationship between the results from the two software. The correlation analysis was performed independently for each of the four experimental variations: tube current, kernel type, task object diameter, and task object contrast.

## 3 RESULTS

### 3.1 TTF and NPS results

**FIGURE 5:**
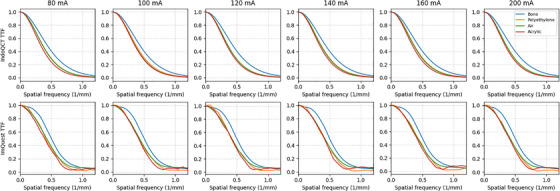
TTF results for tube current variations on the ACR 464 CT phantom image, with bone, polyethylene, air, and acrylic inserts measured using the developed software and ImQuest.

**FIGURE 6:**
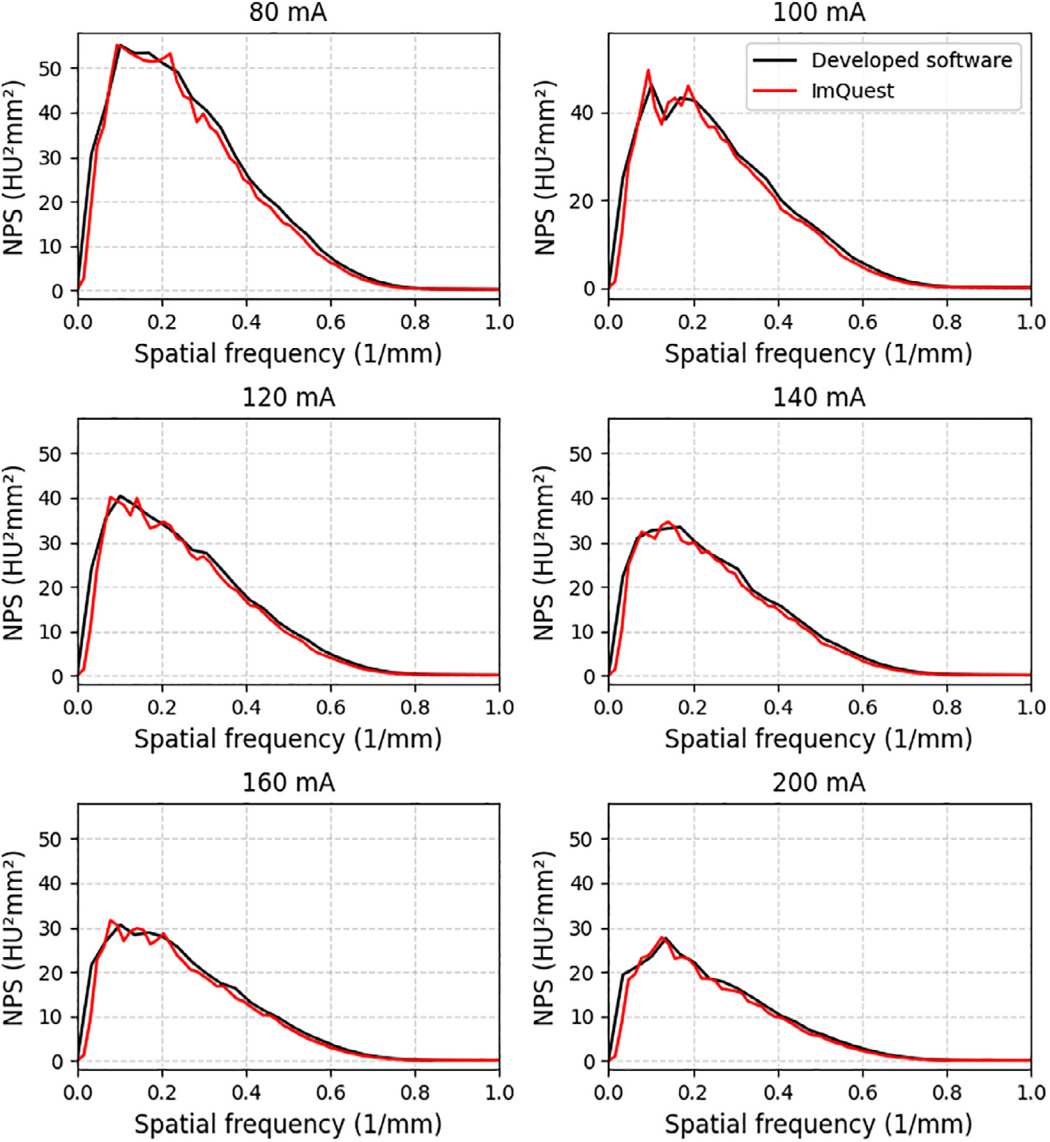
NPS results for tube current variations on the ACR 464 CT phantom image measured using the developed software and ImQuest.

Figures 5 and 6 present the TTF and NPS results of the ACR 464 CT phantom images for tube current variations, respectively. Based on the TTF data, the developed software generally produced curves similar to ImQuest. Resolution differences were observed for each insert. This effect was particularly noticeable for the bone insert, which exhibited a higher response than the others. NPS measurements showed a declining peak trend as the tube current increased. Noise magnitude decreased by 34% and 36% for the developed software and ImQuest measurements, respectively. However, the mean and peak frequency values remain relatively unchanged.

**FIGURE 7:**
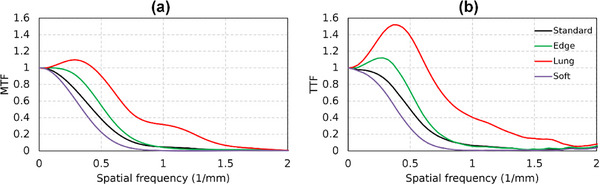
Edge MTF and TTF results of bone insert for kernel type variations on the ACR 464 CT phantom image measured using the developed software (a) and ImQuest (b), respectively.

**FIGURE 8:**
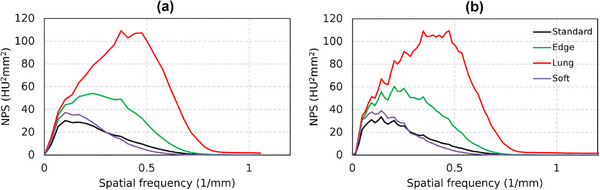
NPS results for kernel type variations on the ACR 464 CT phantom image measured using the developed software (a) and ImQuest (b).

Figures 7 and 8 show TTF and NPS results of the ACR 464 CT phantom images for kernel type variations, respectively. The performance of our software was evaluated against ImQuest by comparing the TTF and NPS for the Standard, Lung, Edge, and Soft kernels. To demonstrate the use of edge‐based MTF for d′ measurements, we obtained edge MTF from phantom edge with nearly 1000 HU contrast (solid water to air). We chose the bone insert for TTF measurement using ImQuest to match the edge contrast. Although the TTF curves show some differences, this is attributed to the different measurement methods: the developed software calculated the MTF from the phantom's edge profile, whereas ImQuest derived the TTF from the bone insert. Despite this methodological difference, the relative spatial resolution performance was highly consistent, with both platforms correctly identifying the kernel hierarchy from the highest to lowest resolution (Lung > Edge > Standard > Soft). The NPS results also demonstrated a very strong agreement. Both methods confirmed that noise magnitude and texture are directly linked to filter sharpness, with nearly identical values for integral NPS and peak frequency. The general curve shapes and peak locations were similar, though the ImQuest results appeared slightly more fluctuating.

### 3.2 Detectability index for tube current variation

**FIGURE 9:**
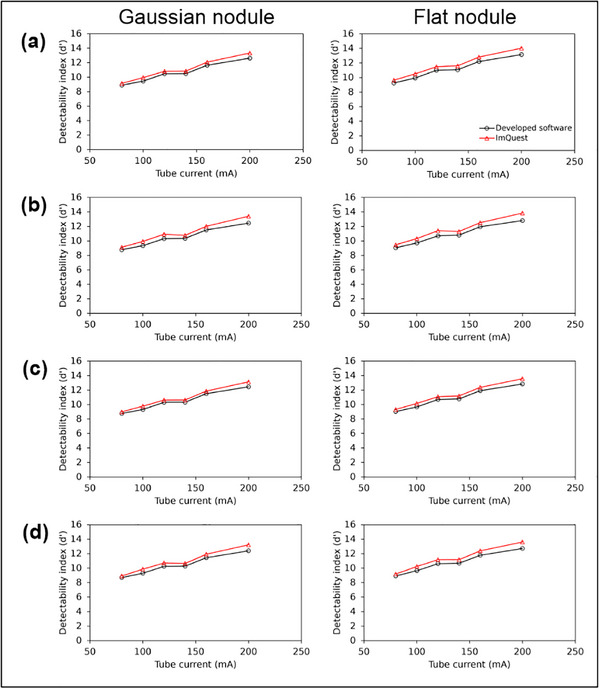
Detectability index (d′) results for tube current variations and two types of nodules (Gaussian and flat). TTF was derived from (a) bone, (b) polyethylene, (c) air, and (d) acrylic inserts.

Figure 9 shows the d′ results for tube currents of 80, 100, 120, 140, 160, and 200 mA, where the TTF was derived from bone, polyethylene, air, and acrylic inserts. In general, across all inserts, the d′ increased as tube current increased. The primary finding is the strong agreement in the performance trend between the two software. As the tube current increases and image noise decreases, both software showed a consistent, predictable increase in the d′ from the NPW detection reading for both Gaussian and flat nodules. This strong concordance is statistically confirmed by a high Pearson correlation coefficient (*r* = 0.98) between the two sets of measurements, although the results of the developed software are systematically slightly lower than those from ImQuest. This measurement bias occurs due to differences in the approaches used for MTF and NPS measurements.

### 3.3 Detectability index for kernel type variation

**FIGURE 10:**
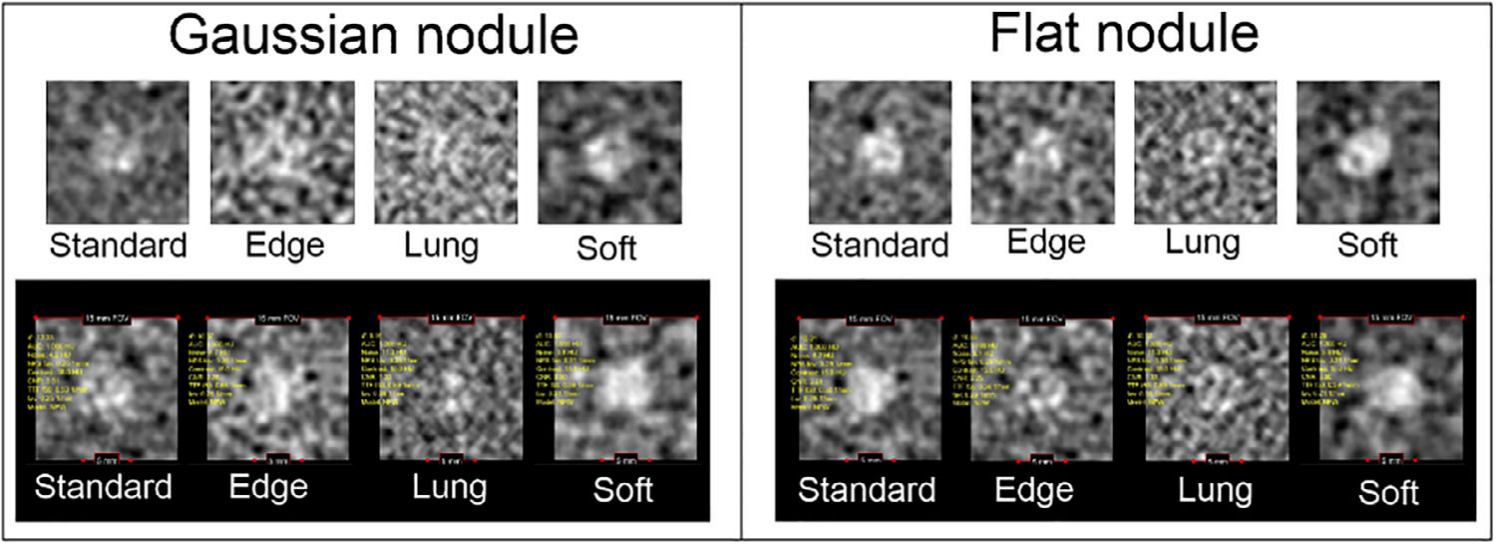
Synthetic images of Gaussian and flat nodules at various kernel types (Standard, Edge, Lung, Soft) generated by the developed software (top) and ImQuest (bottom).

Figure 10 displays synthetic images for various kernel types generated by the developed software and ImQuest for Gaussian and flat nodules. As expected, different noise texture can be clearly observed between different kernel types, with Lung kernel producing the most prominent high frequency noise. Therefore, the task object in the Lung kernel is the most difficult to identify visually. Conversely, the Soft filter produces the most pronounced granular noise, although its level does not significantly interfere with the visibility of the task object. Among the three kernels, the Soft and Standard kernel yield synthetic images in which the object is most easily distinguished from the background. This trend in visual impression is also observed in the d′ images generated by ImQuest.

**FIGURE 11:**
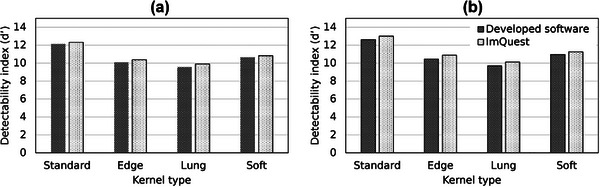
Detectability index (d′) results for kernel type variation measured using the developed software and ImQuest: (a) Gaussian nodule, (b) Flat nodule.

Figure 11 presents the d′ results for kernel type variation in the Gaussian and flat nodules. The calculated d′ values for both the Gaussian (a) and flat (b) nodules demonstrate a strong agreement between the developed software and ImQuest software, with a Pearson correlation *r* > 0.99 for both signal types. A consistent trend was observed across the different kernels, directly correlating with the prior visual assessment. For both nodule types, the Lung kernel consistently yielded the lowest d′ value, hence identifying it as the most challenging condition for object detection. Conversely, Standard kernel produced the highest d′ value, confirming it provided the best object‐to‐background distinguishability.

### 3.4 Detectability index for object diameter variation

**FIGURE 12:**
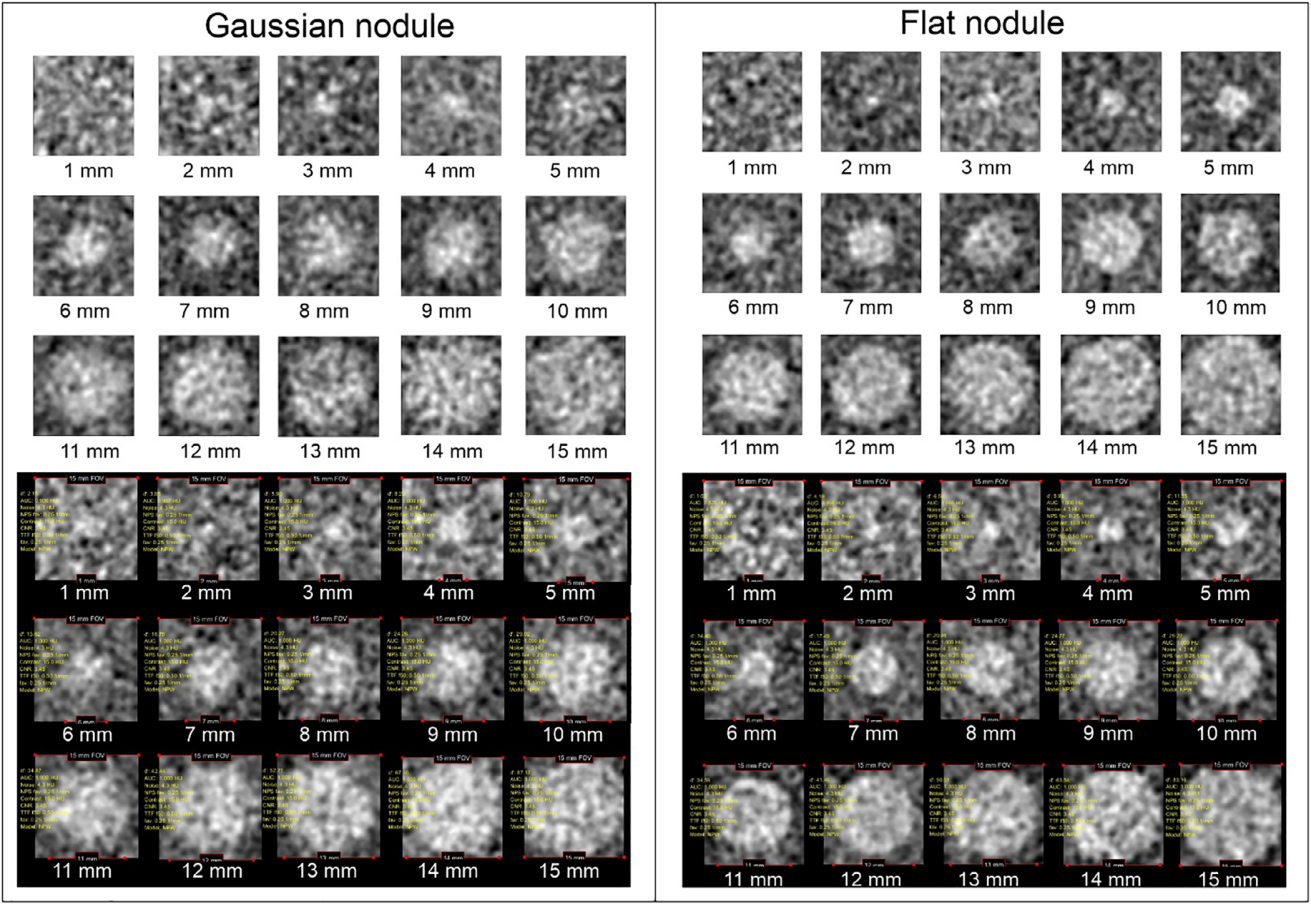
Synthetic images of Gaussian and flat nodules at various object diameters (1–15 mm) generated by the developed software (top) and ImQuest (bottom).

Figure 12 displays synthetic images for various object diameters generated by the developed software and ImQuest for Gaussian and flat nodules. A visual difference is observed between Gaussian and flat nodules. The Gaussian nodule appears more deformed compared to the flat nodule. This phenomenon is seen in both software outputs, indicating that the developed software can replicate results comparable to ImQuest. Additionally, for smaller object diameters (<3 mm), objects became difficult to identify due to low‐frequency noise corruption.

**FIGURE 13:**
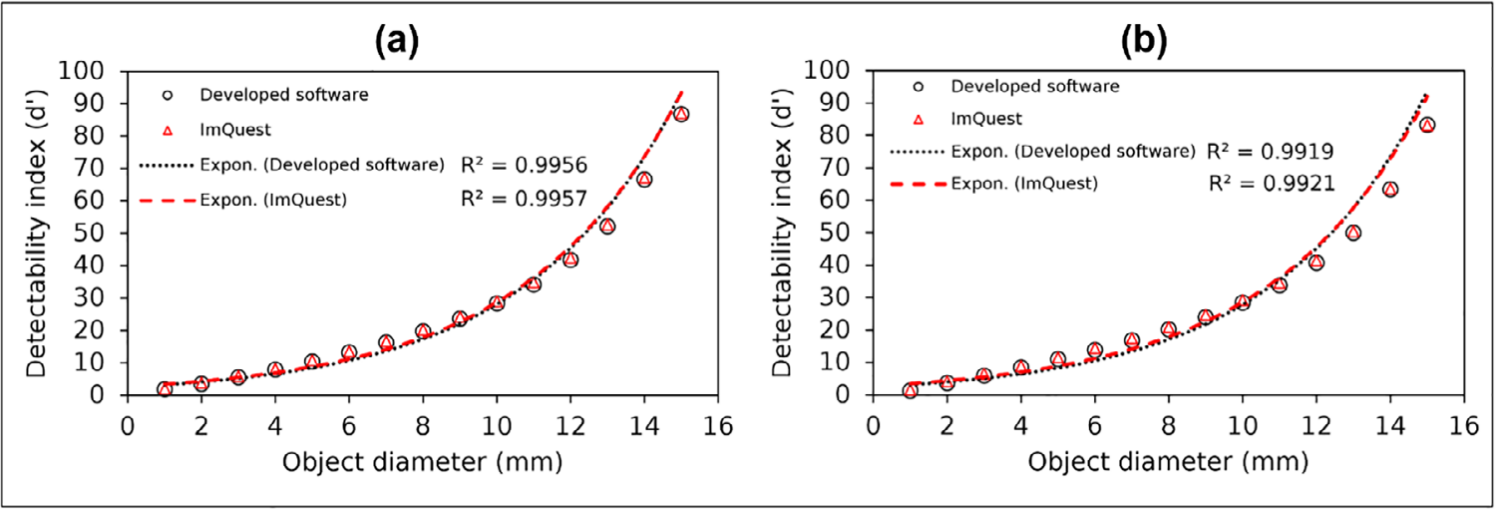
Detectability index (d′) results for object diameter variation measured using the developed software and ImQuest: (a) Gaussian nodule, (b) Flat nodule.

Figure 13 presents the d′ results for object diameter variations in Gaussian and flat nodules. In general, as the object diameter increased, the detectability index followed an exponential trend (*R* ^2^ > 0.99). This suggests that larger objects lead to higher detectability. A Pearson correlation analysis between object diameter variations and measured detectability index values showed a strong agreement (*r* > 0.99) between the developed software and ImQuest for both Gaussian and flat nodules.

### 3.5 Detectability index for object contrast variation

**FIGURE 14:**
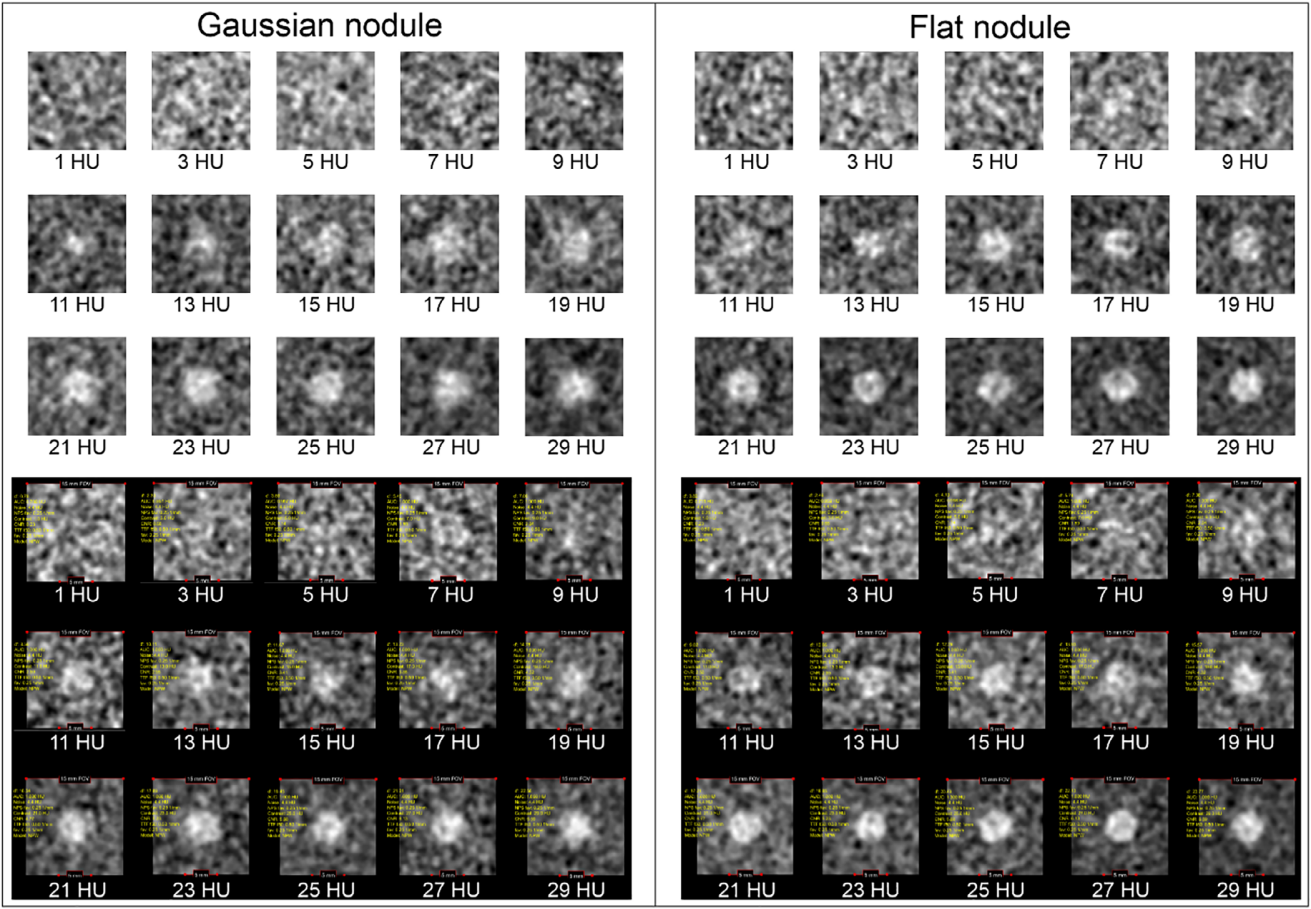
Synthetic images of Gaussian and flat nodules at various object contrast (1‐29 HU) generated by the developed software (top) and ImQuest (bottom).

Figure 14 displays the synthetic images for various object contrasts generated by the developed software and ImQuest for both Gaussian and flat nodules. Generally, at very low contrasts (< 5 HU), the task object is exceedingly difficult to distinguish from the background. This observation is noticeable for both signal types (Gaussian and flat) and is consistent across the results from both the developed software and ImQuest. Objects with a contrast of 7 HU or higher begin to become visually perceptible. It should be noted that the integral of the noise power spectrum (NPS) for the dataset used in the object contrast variation is approximately 4 HU. Consequently, contrasts smaller than or roughly equal to 4 HU are inherently difficult to identify. Additionally, the flat nodule object exhibits a more sharply defined shape compared to the Gaussian nodule, particularly at relatively high contrasts (>15 HU).

**FIGURE 15:**
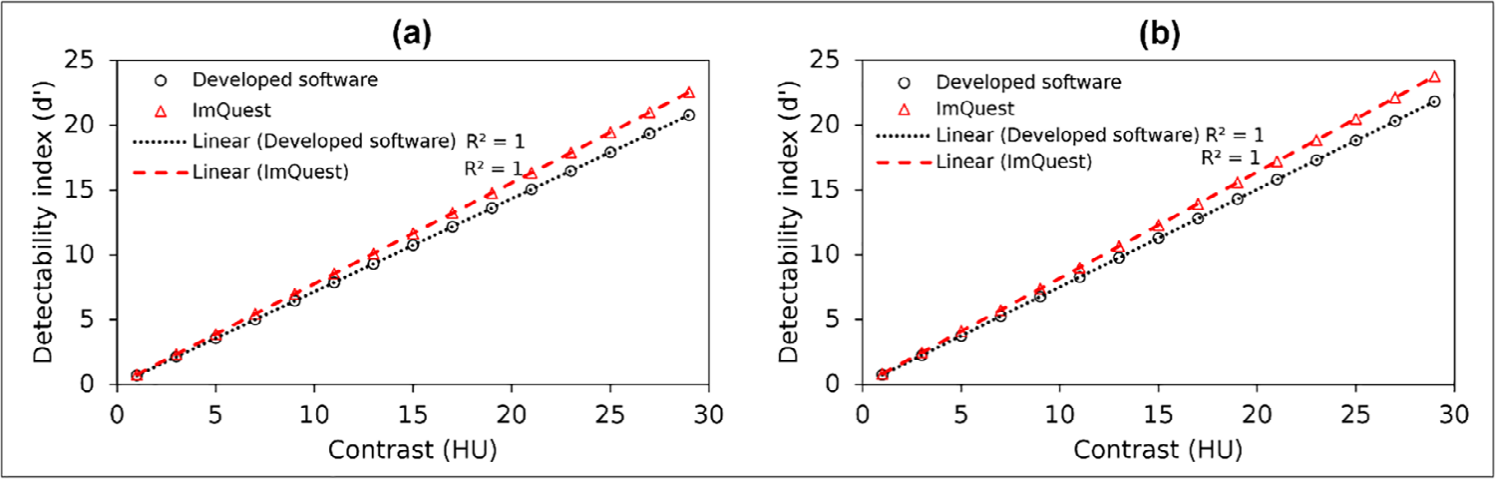
Detectability index (d′) results for object contrast variations measured using the developed software and ImQuest: (a) Gaussian nodule, (b) Flat nodule.

Figure 15 presents the d′ results for object contrast variations in both Gaussian and flat nodules. Based on the measurement data, a higher object contrast leads to a correspondingly higher measured d′, exhibiting a linear relationship (*R* ^2 ^= 1). This trend is observed for both Gaussian and flat nodules in the results generated by the developed software and ImQuest. A Pearson correlation analysis confirmed a strong relationship for both Gaussian and Flat signals (*r* > 0.99). It can also be observed that as the contrast increases, the divergence between the d′ values measured by the two software packages becomes more pronounced, with ImQuest's calculations being consistently higher than those of our developed software.

## 4 DISCUSSION

Characterizing the d′ according to the specific imaging task is crucial for improving diagnostic accuracy. Since d′ measurement involves advanced image quality parameters, specialized software is required. Therefore, we developed a platform designed to help medical physicists to automatically access d′ for optimizing the imaging protocols or studying new imaging systems. This paper aims to introduce d′ measurements using the developed software and validate them by comparing them to ImQuest, an established software. We used ACR 464 CT phantom images acquired at various tube currents, and obtained with various kernel types, along with task function object diameter and contrast variations, to evaluate the trends of d′ results across both software.

It is noted that the developed software has advantages over previous tool: First, the developed software offers automation of ROI placements in TTF and NPS measurements. This reduces subjectivity and speeds up d′ calculations. Second, the developed software integrates this feature with the full range of DICOM viewer features, such as connection to the picture archiving and communication system (PACS) and easier image management. Third, the developed software allows each calculation result to be directly stored in a database. This allows for easier analysis of trends from various d′ measurements over different time periods.

The tube current variation data showed that d′ measured using the developed software closely match ImQuest for both Gaussian and flat nodules. When comparing d′ across different inserts (bone, polyethylene, air, and acrylic), no significant differences were observed. It was found that tube current had a pronounced effect, where higher currents led to an increased d′. This indicates that d′ is highly dependent on noise texture and magnitude, which in this study were modulated through tube current variations.

The d′ results for object diameter variation also showed a strong agreement between the developed software and ImQuest for both Gaussian and flat nodules. As the task function object size increased, the d′ follows an exponential fit (*R* ^2^ > 0.99), with larger objects leading to higher detectability. The minor statistical differences between the developed software and ImQuest were likely due to variability in NPS ROI placement and detrending techniques.

Our software was developed to provide a fully automated workflow for measuring the d′, using the same NPW model observer employed by ImQuest. The primary difference and main advantage of our devloped software compared to the ImQuest is the automation of the initial data extraction steps. Our software implements an algorithm that automatically detects the target object and places the required ROIs for both TTF and NPS measurements. In contrast, the workflow in ImQuest requires the user to manually perform this ROI placement. By automating this critical step, our software is designed to eliminate potential intra‐ and inter‐user variability, thus ensuring more consistent and reproducible measurements from a one‐click operation.

Some fundamental differences can be observed in the TTF curves (Figure 5). A critical consideration here is the methodological difference in TTF calculation, which explains the fundamental variation in curve shape observed between our software and ImQuest. Our software employs a single logistic curve fit to estimate the edge spread function (ESF). This technique is predicated on the assumption that the edge profile is strictly monotonic (i.e., without over‐ or undershoots) and has the benefit of filtering noise from the measurement. Our investigation suggested that single logistic curve fitting is comparable with directly sampling the ESFs and other tools.^23^ This results in a smooth, classic TTF curve shape. In contrast, ImQuest obtaines the TTF more directly from the measured ESF data without enforcing a monotonic fit. Although this allows it to capture non‐monotonic features, it makes the calculation more susceptible to noise. This noise sensitivity can occasionally lead to artifacts, such as false interpolations in the fluctuating tail of the TTF curve.^21^ Therefore, the smoother shape of our TTF is a direct consequence of the curve‐fitting model, whereas ImQuest's TTF provides a more direct representation of the actual edge profile. For the TTF component, if a task‐based phantom is available, users can select the desired insert for measurement. However, if a phantom with multiple inserts is not available, the developed software allows the use of point‐based or edge‐based MTFs as an alternative. The use of edge MTF for calculating the detectability index is demonstrated in the results on kernel type data variations (Figures 7 and 8). The use of MTF in this context requires critical consideration, particularly regarding its limitations in clinical observations. Besides TTF, NPS measurement is designed to be as flexible as possible. Automated ROI placement adapts to shift‐variant characteristics across homogeneous phantom regions. The ROI can be shifted in multiple directions (up, down, left, and right) and can be configured to different quantities (e.g., 1, 2, 3, 4, 5, 6, or 9 ROIs). In this study, we used 5 ROIs shifted to the left and used 5 slices to improve measurement stability. This stability is reflected in the final NPS curves. However, a subtle difference in smoothness can be observed when comparing our results to those from ImQuest. This variation comes from the different computational methods. Each software uses radial averaging method to obtain the 1D NPS from the 2D NPS. Our software employs a denser radial sampling technique, acquiring data points at 1‐degree intervals from the 2D NPS. This approach collected a large number of samples for averaging at each spatial frequency. In contrast, ImQuest uses a rebinning method, where pixels in the 2D frequency domain are grouped into discrete radial bins before averaging. Consequently, the larger number of samples collected by our dense sampling strategy naturally results in a final 1D NPS curve with less variance, appearing smoother than the output from ImQuest.

The software was developed not only to assist in routine quality control and optimization but also to support research and education. To facilitate further analysis, measurement results can be exported in various formats. All measurement curves can be saved as spreadsheet data. Additionally, the synthetic images can be stored in DICOM format, allowing further analysis and broadening the potential for evaluating synthetic images using alternative methods.

Several aspects require further development to enhance task function accuracy. One key area is simulating object shapes based on the pathological conditions being modelled. Currently, the developed software supports only simplified circular shapes with three different task object profiles: Gaussian, flat, and designer. However, the designer task object profile is not evaluated in this study. As a result, object shape information is not yet considered in detectability analysis. The task function modeling in this study simulates an idealized situation, constrained to circular object forms. In clinical practice, lesions frequently show irregular forms that are harder to anticipate. Significant improvements in object shape similarity could be achieved by incorporating realistic shape data into the task function model (e.g. basic non‐circular forms and irregular outlines) and signal position (e.g. displaced and cropped items).

It should be noted that several studies reported that the d′ obtained using the observer model and the human model were comparable.^29^, ^30^ However, in some cases, results using the observer model may not in accordance with the human model results, for example for lesions located in non‐homogeneous areas. It was reported that the same lesion located in a homogeneous and non‐homogeneous area produced a different d′.^31^ This cannot be represented accurately by the observer model.

It should also be noted that the d′ with this method is an approximation. The d′ is calculated based on the TTF value derived from the object on the insert pin within the ACR 464 CT phantom. It is known that the contrast of four insert pins is much greater than the contrast of evaluated task object (i.e. 15 HU). Therefore, the use of TTF from ACR 464 CT phantom to determine the d′ of such low‐contrast task does not actually describe the actual condition of low‐contrast objects. Therefore, this difference must always be considered when the d′ is approximated with this method.

There are other limitations in this study. First, the study is focusing on d' in the ACR 464 CT phantom with a diameter of 200 mm. This may not be applicable to all clinical tasks. Discrepancies are expected between TTF and NPS results derived from the head and body phantoms. It is noted that our goal was to develop and validate an automated d' calculation software in a highly controlled environment. Hence, the ACR 464 CT phantom is ideal for this purpose as it is widely available and allows for reproducible measurements. Second, this study was conducted using only a single CT scanner. Therefore, further studies using images from various CT scanners are needed to obtain more conclusive results. Third, the images used for this study were only reconstructed using filtered‐back projection (FBP). Currently, images are often reconstructed using iterative reconstruction (IR) or deep learning image reconstruction (DLIR), which produce images with non‐linear characteristics. Studying the d′ using these images is important.

## 5 CONCLUSION

Software for automatically measuring the d′ in CT images has been developed. The validity of its results was tested by comparing them with those from the ImQuest, using tube current, kernel type, task function object diameters, and task function object contrasts variations. The results demonstrated a strong agreement between the developed software and ImQuest across test conditions. Furthermore, the developed software provides an intuitive and straightforward workflow, allowing users to easily and automatically access d′ measurements.

## AUTHOR CONTRIBUTIONS

**Choirul Anam**: Writing—review & editing; validation; supervision; software; resources; project administration; methodology; investigation; funding acquisition; formal analysis; data curation; conceptualization. **Ariij Naufal**: Writing—original draft; software development; validation; supervision; resources; methodology; investigation; formal analysis; data curation. **Zaenal Arifin**: Writing—review & editing; investigation; formal analysis; data curation. **Eko Hidayanto**: Writing—review & editing; investigation; formal analysis; data curation. **Evi Setiawati**: Writing—review & editing; investigation; formal analysis; data curation. **Fajar Arianto**: Writing—review & editing; investigation; formal analysis; data curation. **Toshioh Fujibuchi**: Writing—review & editing; validation; supervision. **Geoff Dougherty**: Writing—review & editing; validation; supervision; methodology.

## CONFLICT OF INTEREST STATEMENT

Choirul Anam, Ariij Naufal, and Geoff Dougherty are developers of IndoQCT. Other authors declare that they have no conflict of interest.

## ETHICS STATEMENT

This article does not contain any studies with human participants or animals performed by any of the authors.

## Data Availability

All data that support the findings of this study are included within the article (and any supporting information files).
